# Prognostic effect of atrial fibrillation on survival in patients with hypertrophic cardiomyopathy: a meta-analysis

**DOI:** 10.1186/s13019-023-02299-x

**Published:** 2023-06-20

**Authors:** Meiling Du, Xiaoyuan Wang, Aiai Zhang, Feixing Li, Mengyang Yi

**Affiliations:** Department of Cardiology, The First Affiliated Hospital of Hebei North University, No. 12 of Changqing Road, Qiaoxi District, Zhangjiakou, 075000 China

**Keywords:** Atrial fibrillation, Hypertrophic cardiomyopathy, Prognosis results

## Abstract

**Objective:**

To systematically evaluate the prognostic impact of atrial fibrillation (AF) in patients with hypertrophic cardiomyopathy (HCM).

**Methods:**

The Chinese and English databases (PubMed, EMBASE, Cochrane Library, Chinese National Knowledge Infrastructure, and Wanfang database were systematically searched to include observational studies on the prognosis of AF in cardiovascular events or death in patients with HCM; these were evaluated using Revman 5.3.

**Results:**

After systematic search and screening, a total of 11 studies with a high study quality were included in this study. Meta-analysis showed that patients with HCM accompanied by AF had a higher risk of all-cause death (odds ratio [OR] = 2.75; 95% confidence interval [CI]: 2.18–3.47; *P* < 0.001), heart-related death (OR = 2.62; 95%CI: 2.02–3.40; *P* < 0.001), sudden cardiac death (OR = 7.09; 95%CI: 5.77–8.70; *P* < 0.001), heart-failure-related death (OR = 2.04; 95%CI: 1.24–3.36; *P* = 0.005), and stroke death (OR = 17.05; 95%CI: 6.99–41.58; *P* < 0.001) compared with patients with HCM without AF.

**Conclusion:**

Atrial fibrillation is a risk factor for adverse survival outcomes in patients with HCM, and aggressive interventions are needed in this population to avoid the occurrence of adverse outcomes.

## Introduction

Hypertrophic cardiomyopathy (HCM) is the most common form of inherited cardiomyopathy and is mainly characterised by the thickening of the left ventricular folds, with an overall incidence of 0.2% (1 in 500 people are affected) [1, 2]. The disease is often associated with many types of arrhythmia, such as atrial arrhythmias and ventricular arrhythmias, of which atrial fibrillation (AF) is the most common arrhythmia occurring in HCM. Epidemiological data showed a prevalence of HCM with AF of 10–30% [3, 4], and a 33-item based meta-analysis showed an overall prevalence of AF in patients with HCM of 22.45% [5].

Previous studies have shown a clear association between AF and poor prognostic outcomes in patients with HCM. The occurrence of AF has a significant impact on the quality of life of patients with HCM and is often associated with decreased function. Atrial fibrillation is significantly associated with the occurrence of heart failure, thromboembolism, and death [6]. Studies have found a 27.1% incidence of thromboembolism in patients with HCM accompanied by AF [5]. Compared with the sinus rhythm, the occurrence of AF is associated with a 4× higher risk of death [6]. A follow-up study of 480 patients with HCM found that 107 patients developed AF during a mean follow-up period of 9.1 years; the mortality rate (3% vs. 1%) was significantly higher than in patients without AF [7].

Currently, there are no randomised clinical trials on HCM in patients with AF. Most of the previously published meta-analyses assessed the incidence of AF in patients with HCM in regard to the size of the left atrium and the patient’s risk of thromboembolism [8]. Meanwhile, due to the wide research scope and selection criteria of these analyses, there are few analyses that report mortality results. Therefore, the aim of this study is to systematically analyse the effects of AF on all-cause mortality, cardiac-related mortality, and stroke mortality in patients with HCM and to gain insight into the impact of survival prognosis in patients with HCM and AF.

## Materials and methods

### Search strategy

Following the PRISMA guidebook, a systematic literature search of PubMed, Embase, Cochrane Library, Web of Science, CINAHL, Chinese National Knowledge Infrastructure, and the Wanfang database was performed from the date of inception of the databases to May 30, 2022. A search strategy combining subject headings and free words was used. The search terms included ‘hypertrophic cardiomyopathy’ or ‘HCM’, ‘atrial fibrillation’ or ‘AF’, ‘survival’, or ‘mortality’. The target literature was obtained by reading the relevant systematic reviews.

### Inclusion and exclusion criteria

Inclusion criteria: (1) Studies published in peer-reviewed journals in English and Chinese; (2) patients diagnosed with HCM with or without AF; and (3) patients with a primary outcome measure of all-cause mortality and secondary outcome measures of heart failure mortality, sudden cardiac death mortality, stroke mortality, and cardiac-related mortality.

Exclusion criteria: (1) non-population studies; (2) conference articles, case reports, systematic reviews, and other research types; (3) studies with insufficient outcome information that could not be analysed; (4) repeated reports of literature research; and (5) studies for which complete articles could not be obtained.

### Study selection and data extraction

Two reviewers independently reviewed the abstracts and full texts of each article according to the inclusion and exclusion criteria. For disagreements between the two reviewers, a third reviewer was recruited for discussion until a consensus was achieved. After literature screening, two reviewers both independently extracted the following information: literature information, demographic characteristics of the subjects, diagnosis mode of AF, and outcome measures (e.g. all-cause mortality, heart failure mortality, sudden cardiac death mortality, stroke mortality, and cardiac-related mortality).

### Quality evaluation

The Newcastle–Ottawa Literature Quality Assessment Scale (NOS) was used to evaluate the quality of observational studies. The scale was evaluated using eight items: (1) the method of selecting the non-exposure group; (2) the method of determining exposure factors; (3) the presence or absence of outcome indicators; (4) comparability between groups; (5) the adequacy of the study’s evaluation of outcomes; (6) the adequacy of the follow-up period; and (7) the completeness of the follow-up. The full score was 9 points. A total score of 7 points or more equalled high-quality literature, and 5 points or less equalled low-quality article.

### Statistical analysis

Statistical analysis was performed using the Revman 5.3 software. The measurement results selected in this study were all categorical variables. The Mantel–Haenszel method was used to evaluate the comprehensive situation of each clinical endpoint in each study. The results of the meta-analysis were expressed by the odds ratio (OR) and the corresponding 95% confidence interval (CI). The heterogeneity test was used to determine the size of heterogeneity by the test of ${I}^{2}$. If ${I}^{2}$ < 50% or *P* > 0.1, the included literature was considered homogeneous, and the fixed effect model (Mantel–Haenszel) was used for analysis; if ${I}^{2}$ > 50% or *P* ≤ 0.1, the included studies were considered heterogeneous, and the random effect model (DerSimonian–Laird) was used for analysis. If the heterogeneity was large, a sensitivity analysis was used to explore the source of heterogeneity. A *P* value of < 0.05 indicated that the difference was statistically significant.

## Results

### Study characteristics

**Fig. 1:**
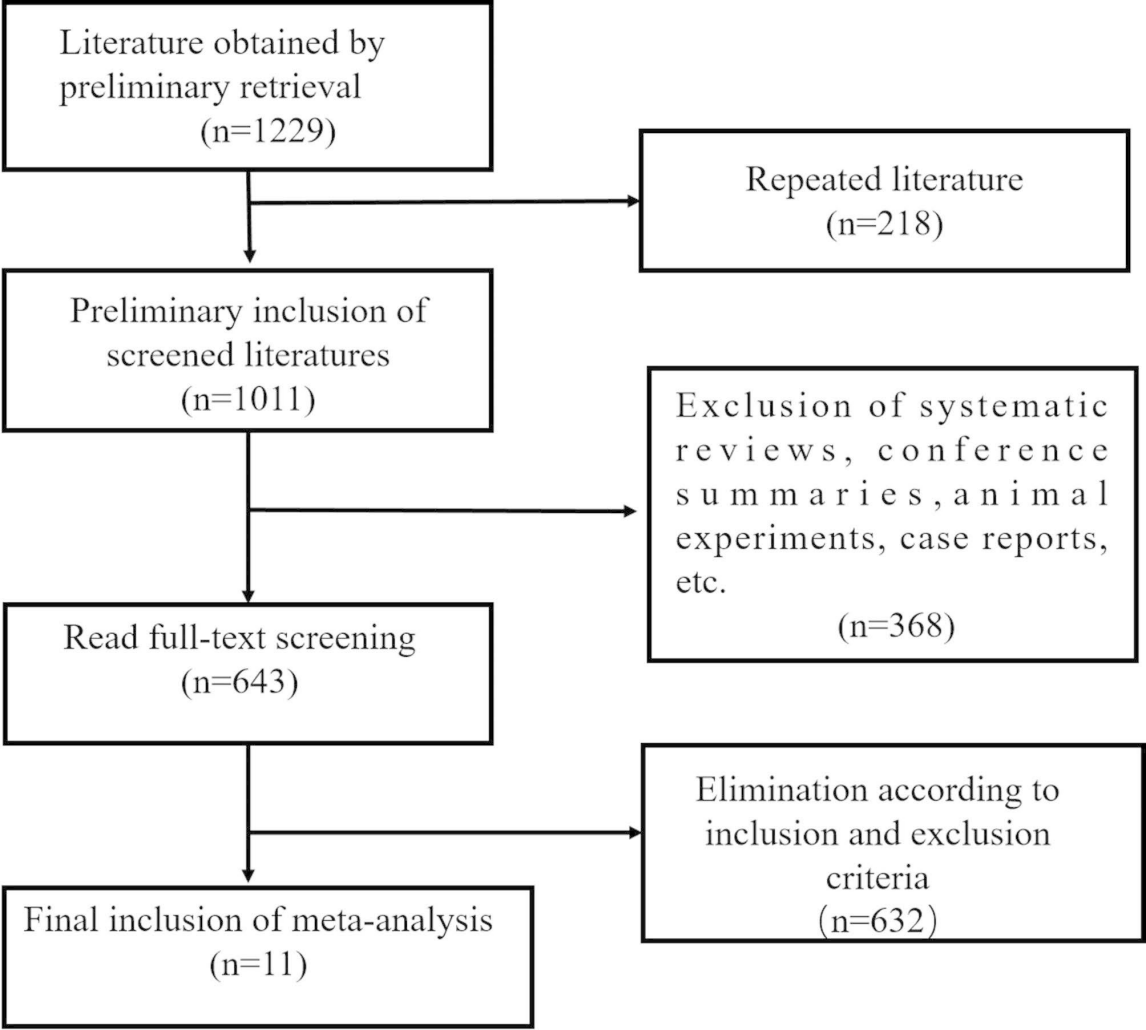
Flow chart of literature selection

In this study, after systematic searching and screening of Chinese and English databases, 11 studies [7, 9–18] exploring the survival prognosis results of AF in patients with HCM were included in the final meta-analysis. The flow chart of literature searching and screening is shown in Fig. 1. Eleven studies were published in 1990–2021 (four from the US, three from China, two from Korea, and one from Japan, Italy, and England). Four studies were case-control studies, three were prospective cohort studies, and four were retrospective cohort studies. The follow-up was 0.5 years, with the longest being 9.1 years. Eleven studies included 27,369 patients with HCM, of which 3,913 had AF, and nine studies used electrocardiograms for AF testing. The included studies had a high literature quality, with NOS scores of 6–9 (average score of 7.51). Basic characteristics of more included studies 1. as shown as Table 1.

### All-cause mortality

**Fig. 2:**
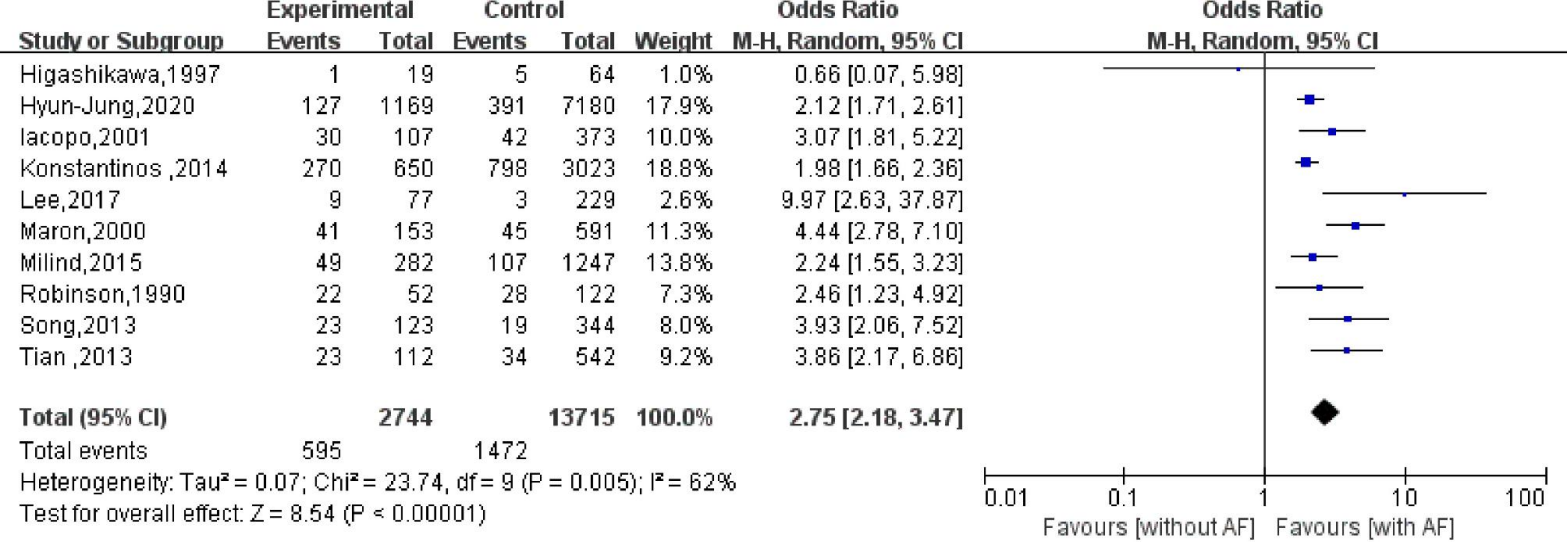
Effect of atrial fibrillation on all-cause mortality in patients with hypertrophic cardiomyopathy

A total of 10 studies have reported the impact of AF on all-cause mortality in patients with HCM. Among these studies, 2,744 patients with AF died, while 13,715 patients without AF died. The heterogeneity evaluation results showed that there was some heterogeneity in the included studies (${I}^{2}=62\%$; *P* = 0.005), and the random-effects model was used to calculate the pooled statistics. Meta-analysis showed that AF increased all-cause mortality in patients with HCM compared with those without AF, with a pooled effect size of OR = 2.75 (95%CI: 2.18–3.47; *P* < 0.001; Fig. 2). After exclusion of one study [13], the sensitivity analysis showed that heterogeneity among the included studies was reduced by 12%, and AF still increased all-cause mortality in patients with HCM (OR = 2.22; 95%CI: 1.98–2.50; *P* < 0.001).

### Heart-related death

**Fig. 3:**
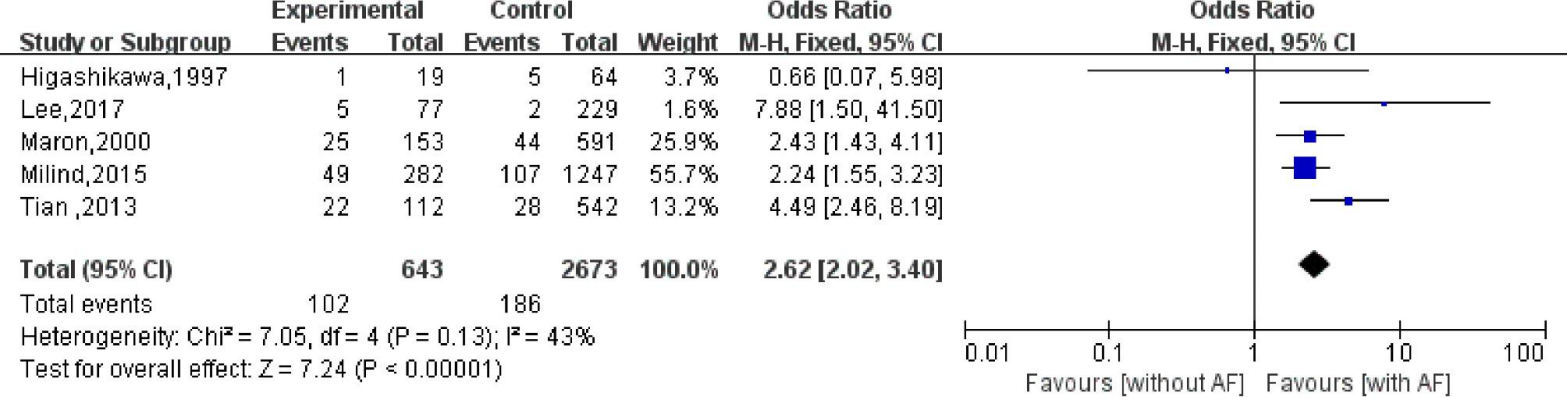
Effect of atrial fibrillation on cardiac-related death in patients with hypertrophic cardiomyopathy

A total of five studies have reported the impact of AF on cardiac-related mortality in patients with HCM. Among these studies, 643 patients with AF died from cardiac-related causes, while 2,673 patients without AF died from cardiac-related causes. The heterogeneity evaluation result was *I*^2^ = 43% (*P* = 0.13), suggesting that there was little heterogeneity among the included studies. The fixed-effect model was used for meta-analysis. The results of the analysis showed that patients with HCM accompanied by AF had a 2.62× higher risk of cardiac-related death than those without AF (95%CI: 2.02–3.40; *P* < 0.001; Fig. 3).

### Sudden cardiac death

**Fig. 4:**
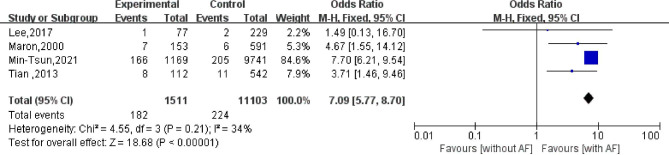
Effect of atrial fibrillation on sudden cardiac death in patients with hypertrophic cardiomyopathy

Four studies have reported the impact of AF on sudden cardiac death in patients with HCM. Among these studies, 1,511 patients with AF experienced sudden cardiac death, while 2,673 patients without AF experienced sudden cardiac death. The results of the heterogeneity evaluation (${I}^{2}=34\%$; *P* = 0.21) suggested the use of the fixed effects model for systematic evaluation. Compared with patients without AF, those with AF had an increased risk of sudden cardiac death when accompanied by HCM (OR = 7.09, 95%CI: 5.77–8.70; *P* < 0.001; Fig. 4).

### Heart-failure-related death

**Fig. 5:**
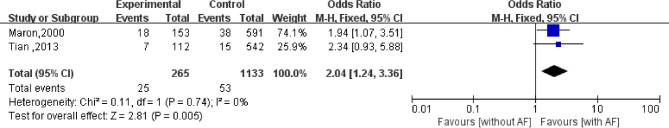
Effect of atrial fibrillation on heart failure-related death in patients with hypertrophic cardiomyopathy

Two studies have reported the impact of AF on heart-failure-related mortality in patients with HCM. Among these studies, 285 patients with AF died from heart failure, while 1,133 patients without AF died from heart failure. The heterogeneity test result (${I}^{2}=0\%$; *P* = 0.74) indicated good homogeneity among the included studies, and the fixed utility model was used to calculate the pooled effect size. Compared to patients with HCM without AF, patients with HCM and AF had a risk of heart-failure-related death, with a pooled effect size of 2.04 (95%CI: 1.24–3.36; *P* = 0.005; Fig. 5).

### Stroke death

**Fig. 6:**
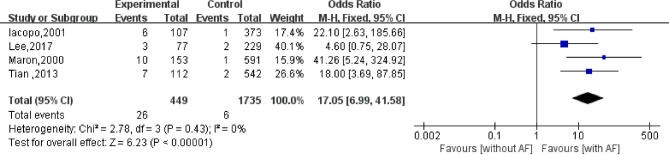
Effect of atrial fibrillation on stroke mortality in patients with hypertrophic cardiomyopathy

Four studies have reported the impact of AF on stroke mortality in patients with HCM. Among these studies, 449 patients with AF died from stroke, while 1,735 patients without AF died from stroke. The results of the heterogeneity evaluation (${I}^{2}=0\%$; *P* = 0.43) showed low heterogeneity among the included studies. The pooled effect size was calculated using a fixed-effect model. Patients with HCM accompanied by AF had a 17.05 × (95%CI: 6.99–41.58) higher risk of stroke death than those without AF; the difference was statistically significant (*P* < 0.001; Fig. 6).

### Publication bias

**Fig. 7:**
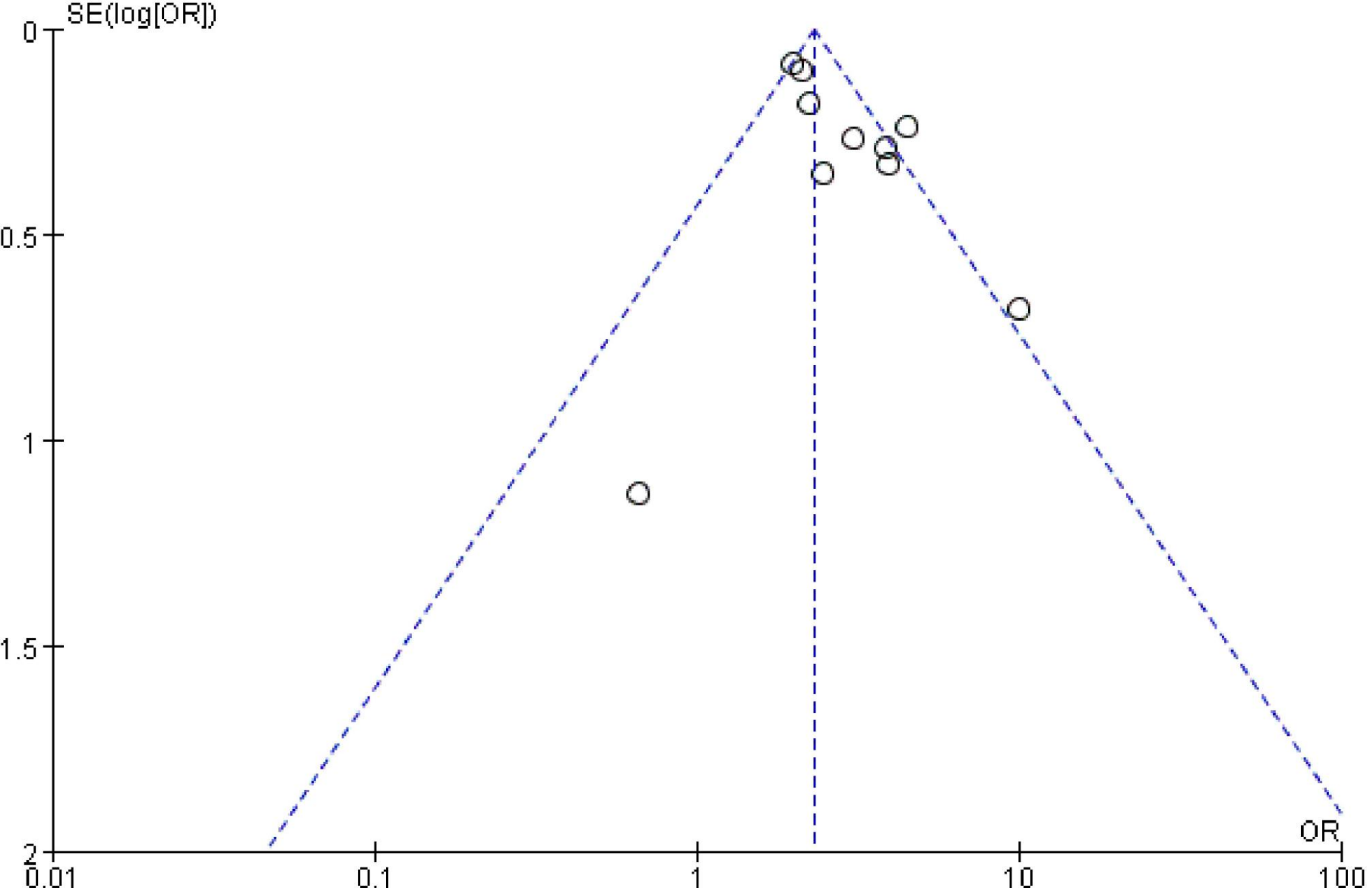
Funnel plot of publication bias for studies reporting all-cause mortality

The publication bias of the included studies was evaluated based on 10 studies reporting all-cause mortality. The funnel plot (Fig. 7) showed that the scatter was mainly concentrated above the funnel plot and that the distribution was uniform and symmetrical, suggesting that the publication bias between the included studies was small and had some acceptability.

**Table 1:** Basic characteristics of included studies and literature quality evaluation table

study	country	Study type	Follow-up time(year)	Diagnosis mode of atrial fibrillation	Sample Size(AF+/AF-)	age(y)	male(%)	NOS score
Min-Tsun,2021	China,Taiwan	Case-control study	8.5	ICD-9CM	1169/9741	62 ± 13	52.5	8
Robinson,1990	UK	Retrospective cohort study	7	physical examination、dynamic electrocardiogram	52/122	NR	55.7	6
Konstantinos, 2014	USA	Retrospective cohort study	6.1	electrocardiogram	650/3023	55 ± 16	55	7
Tian, 2013	China	Case-control study	4.2	ultrasound cardiogram	112/542	50 ± 15	71	8
Maron,2000	USA	Prospective cohort study	8	ultrasound cardiogram	153/591	NR	62	8
Lee,2017	Korea	Retrospective cohort study	5.5	ultrasound cardiogram	77/229	62 ± 11	NR	8
Iacopo,2001	Italy/USA	Prospective cohort study	9.1	electrocardiogram	107/373	55 ± 15	NR	8
Higashikawa,1997	Japan	Retrospective cohort study	5	ultrasound cardiogram	19/64	50.3 ± 13.9/55.5 ± 7.8	72.3	6
Milind,2015	USA	Prospective cohort study	8.1	ultrasound cardiogram	282/1247	50 ± 13	63	7
Song,2013	China	Case-control study	0.5–0.8	ultrasound cardiogram	123/344	51.8 ± 13.1/48.5 ± 16.0	56	8
Hyun-Jung,2020	Korea	Case-control study	2.5	ICD-10CM	1169/7180	60.7 ± 11.9	69.2	9

AF+: patients with atrial fibrillation; AF-: patients without atrial fibrillation; NOS: Newcastle-Ottawa Scale; NR: not reported; ICD-9CM: International Classification of Disease Ninth Revision Clinical Modification codes; ICD-10CM: International Classification of Disease Tenth Revision Clinical Modification codes; NOS: Newcastle-Ottawa Scale

## Discussion

The results of this study were based on research from Chinese and English databases, and 11 studies evaluating the prognostic results of AF on the survival of patients with HCM were included in the final meta-analysis. The results of this study suggest that AF is a risk factor for poor survival outcome in patients with HCM. Compared with patients with HCM without AF, patients with HCM and AF had a 2.75× higher risk of all-cause mortality, a 2.62× higher risk of cardiac-related death, a 7.09× higher risk of sudden cardiac death, a 2.04× higher risk of heart-failure-related death and a 17.05× higher risk of stroke death. Previous findings suggest that AF is a valid predictor of adverse survival outcomes in patients with HCM. Desai et al. [16]. showed that the presence of AF increased the risk of sudden death and implantable cardioverter defibrillator intervention in patients with HCM after multivariate analysis (HR = 1.90; 95%CI: 1.32–2.72). In addition, Lee et al. [17]. showed a significant association between AF and all-cause mortality in patients with HCM (HR = 1.48; 95%CI: 1.27–1.71) after adjusting for age, gender, and multiple confounders of sudden cardiac death.

The occurrence of AF may be the cause of the combined effect of left atrial structural remodelling and electrical remodelling caused by HCM and may also be associated with genetic factors. Patients with HCM and AF have reduced left ventricular compliance due to left ventricular hypertrophy, increased left ventricular end-diastolic pressure leading to increased left atrial afterload and progressive enlargement of the left atrium, which in turn leads to secondary left atrial cardiomyopathy [19] (which is more likely to lead to the occurrence of AF) [20, 21]. Left atrial electrical remodelling also has an important impact on the occurrence of AF, and in patients with HCM and AF, the *P* wave duration and *P* wave dispersion are predictors of the occurrence of AF. Studies have shown that for patients with HCM accompanied by AF with a P wave duration of > 140 ms under sinus rhythm, the risk of future AF is significantly higher, with a sensitivity and specificity of 56% and 83%, respectively [22]. A P wave dispersion of > 52.5 ms achieves a sensitivity and specificity of 96% and 91%, respectively, in differentiating HCM from AF [23]. Therefore, long-term increased left ventricular filling pressure in HCM causes secondary atrial cardiomyopathy, which makes atrial contraction asynchronous and prolongs the P wave duration, leading to the occurrence of AF. In addition, Tuluce et al. [24]. showed that polymorphisms in the angiotensin receptor gene *AGTR1* are associated with the development of HCM with AF. A study on people with hypertension found that the aldosterone-secreting CYP11B2-344T > C polymorphism was also associated with the development of AF [25]. Therefore, the renin–angiotensin–aldosterone system genes may play a role in the development of AF in patients with HCM.

Atrial fibrillation can further reduce the cardiac function of patients with HCM. Theoretically, once AF occurs, the atrium will lose its effective systolic function, further aggravate the original atrial congestion, further increase the internal pressure of the atrium, and gradually expand the atrium. According to the Frank Starling law, the atrial myocardial contractility will further decline, and the ventricular end diastolic filling will be further reduced. Thus, the cardiac output reduces, decreasing the patient’s cardiac function status [5]. The heart function of patients with AF in New York Heart Association (NYNA) class III–IV was significantly higher than that of patients with a sinus rhythm. An observational analysis of 261 patients from nine hospitals published by Gaita F et al. [26]. showed that a total of 74 patients with HCM with paroxysmal or permanent AF were included; among them, cardiac function was found in 17 patients with HCM accompanied by AF in NYNA Class III–IV, whereas in patients with HCM without AF, overall function was maintained in good condition; however, one year after the onset of AF, 80% of the patients were readmitted because of cardiac insufficiency.

This study has some limitations. First, there was some heterogeneity among the included studies due to the differences in the study population and follow-up time. In addition, because the currently published relevant studies are observational studies, and there is a lack of clinical randomised controlled trials, only observational studies were systematically evaluated. Finally, due to the paucity of relevant literature currently published, only 11 target articles were analysed.

In summary, AF has a significant negative effect on the survival prognosis of patients with HCM. Patients with HCM accompanied by AF have a higher all-cause mortality rate, and AF not only causes failure by increasing the burden on the heart but also leads to an increased risk of heart-related death and sudden cardiac death. In addition, AF significantly increases stroke mortality in patients with HCM. Due to some limitations of this study, future prospective large studies based on different populations are needed to confirm the present conclusions.

## Data Availability

All data generated or analyzed during this study are included in this article.
